# Empathy Modulates the Effect of Stress Reactivity on Generous Giving

**DOI:** 10.3389/fnins.2022.814789

**Published:** 2022-04-25

**Authors:** Hagar Azulay, Nitzan Guy, Yoni Pertzov, Salomon Israel

**Affiliations:** ^1^Department of Psychology, The Hebrew University of Jerusalem, Jerusalem, Israel; ^2^Department of Cognitive and Brain Sciences, The Hebrew University of Jerusalem, Jerusalem, Israel; ^3^Scheinfeld Center of Human Genetics for the Social Sciences, The Hebrew University of Jerusalem, Jerusalem, Israel

**Keywords:** stress, trier social stress test, cortisol, dictator game, generosity, giving, empathy, prosocial

## Abstract

How does acute stress influence the degree to which we cooperate with others? Research on the effects of stress on social decision-making is guided by two seemingly contrasting theories. Acute stress may trigger a Fight-or-Flight response, manifested by increased anxiety, and more egocentric or selfish behavior. Alternatively, according to the Tend-and-Befriend model, acute stress may induce affiliative behaviors, marked by increased prosociality in an effort to seek and receive social support and protection. Extant studies on the topic do not provide consistent support for either pattern of behavior, with studies showing evidence for both Fight-or-Flight or Tend-and-Befriend like responses. One possibility, may be the nature of social responses to stressful situations differ as a function of the individual. In the current study, we demonstrate an example of such a person-by-situation interaction, showing that acute stress can cause either pro-social or selfish responses, contingent on individual differences in trait empathy. One hundred and twenty three participants (60 F) were assessed for trait empathy using the Interpersonal Reactivity Index; consequently, they underwent either the Trier Social Stress Test—a well-validated paradigm for eliciting acute psychosocial stress—or a non-stress inducing control condition. Following exposure to either the stress or control condition, participants played a one-shot Dictator Game to evaluate their generosity levels. Statistical analyses revealed that acute stress by itself did not affect the amount transferred in the Dictator Game. Rather, individual differences in trait empathy moderated the effects of stress on giving. Elevations in stress-induced cortisol resulted in more generous behavior, but only in individuals high in empathy. In contrast, in individuals low in empathy, a greater rise in stress-induced cortisol resulted in more selfish behavior. Effects were more pronounced in females than males. Our findings highlight the necessity of integrating personality traits as important moderators of the link between stress and sociality.

## Introduction

Human social interactions show considerable variation in prosocial behaviors—actions intended to benefit the welfare of others. Understanding the contexts and sources of such heterogeneity, including the biological processes which give rise to them, is a key objective of evolutionary psychology (Henrich and Muthukrishna, 2021). In the current paper, we examine the role of acute psychosocial stress in modulating the preference to share with others.

Stress shapes human’s life by directing behavior, cognition, emotion and health (McEwen, 1998). Day-to-day situations, such as being stranded in an elevator or speaking in public, trigger the acute stress response. The nature of this stress response relies on immediate short-term effects, such as increases in heart rate and blood glucose, guided by the sympathetic-adrenal axis; and on longer-lasting neuroendocrine responses guided by the Hypothalamic-Pituitary-Adrenal (HPA) axis. This axis operates by the hypothalamic release of corticotropin-releasing factor (CRF), leading to release of adrenocorticotropic hormone (ACTH), and ultimately bringing forth the secretion of the steroid hormone cortisol, a key regulator of the stress response (Wadsworth et al., 2019). These combined processes allow for resources to be mobilized to ensure adaptive coping, and underlies necessary behavioral responses to stressors. A sharp increase in cortisol levels is therefore considered a reliable biomarker of stress.

While the physiological response to acute psychosocial stress is, by now, well-characterized, less is known regarding the effects of stress on social decision making. Theoretical models regarding the social response to stress present two seemingly conflicting biobehavioral strategies. According to the classic “Fight or Flight” pattern [now updated to Fight, Flight, or Freeze; Roelofs (2017)], stress should precipitate more antisocial patterns of behavior, reflected in self-preserving actions aimed at fighting the stressor or escaping it (Cannon, 1932). In contrast, the complementary “Tend and Befriend” model, posits that stress can engender affiliative responses by which people turn to each other for protection and support (Taylor et al., 2000; von Dawans et al., 2012).

A typical design of studies in the field assessing these competing theories is to follow-up acute stress exposure with a behavioral decision-making task used to assess prosocial behavior. For example, in the widely used Dictator Game (Kahneman et al., 1986), participants decide how much money, if any, they want to share with another person. To ensure that giving in the game is based on prosocial motivation, rather than other more strategic considerations, the game is designed as one-shot and players identities are kept anonymous.

The empirical record regarding the effects of acute stress on Dictator giving is quite mixed. While some studies have shown that acute stress increases prosocial behavior (von Dawans et al., 2012, 2019; Margittai et al., 2015; Domes and Zimmer, 2019), others show that stress decreases it (Vinkers et al., 2013), with others still showing no effect of acute stress on social decision making (Steinbeis et al., 2015; Veszteg et al., 2021). Consequently, the emerging consensus is that the effects of stress on social behavior are not well-described by simple main effects, and likely are dependent on additional factors [see reviews in Buchanan and Preston (2014) and von Dawans et al. (2021)], such as the type of decision and it’s timing following stress exposure. The current work focuses on the potential contribution of individual characteristics such as sex and personality to the phenomenon.

Sex is a key factor underlying differences in stress reactivity and its effects on social behavior. The Tend and Befriend theory was initially postulated as a female-specific response to stress (Taylor et al., 2000; Taylor, 2006), whereby the female oxytocinergic system directs prosocial behaviors to ensure protection by others, and therefore increases the chances of survival for self and offspring. While studies have largely corroborated this behavioral pattern (Youssef et al., 2012, 2018; Nickels et al., 2017), additional studies have also found evidence consistent with Tend and Befriend-like responses in males as well (von Dawans et al., 2012), so there are still open questions regarding the degree by which differences in social responses to stress may be sex-specific.

*Empathy*—the “sharing and understanding [of another person’s] affective states” (Zaki, 2014, p. 1,608)—is thought to regulate prosocial behavior. According to the empathy-altruism hypothesis, empathic concern for others produces a motivational state aimed at improving the their welfare (Batson et al., 2015). Along these lines, inducing empathic concern experimentally results in greater levels of generosity in social decision-making paradigms (Klimecki et al., 2016). Similarly, individuals higher in trait empathy, i.e., a higher capacity for empathic reactions is a stable feature of their personality (Wallmark et al., 2018), tend to also be more prosocial (Kamas and Preston, 2021). Notably, however, researchers have raised attention to several cases by which the connection between empathy and altruistic behavior are less reliable, calling for more research to understand the specific contexts in which empathic concern facilitates prosocial behavior (Decety, 2021).

One framework potentially accounting for the interactive effects of stress and empathy on social decision making, is the stress induced deliberation-to-intuition (SIDI) model (Yu, 2016). The model postulates that via its effects on connectivity between the amygdala and pre-frontal regions, stress heightens intuitive responses over more deliberative thinking. Notably, such stress-induced shifts toward more gut-based judgements are critically dependent on contextual factors and individual differences (Lighthall et al., 2012). In the case of empathy and social decision-making, stress would potentiate more prosocial decisions in highly empathetic individuals, and vice versa—more selfish decisions in individuals lacking empathy.

The current study aimed to contribute to this growing field of research by examining the role of individual differences in empathy in modulating social responses to stress. Participants (*n* = 123, 60 F) underwent either the Trier Social Stress Test (TSST; Kirschbaum et al., 1993), an extensively validated paradigm for inducing psychosocial stress, or a non-stressful control condition. Following exposure to stress or control, participants’ social preferences were assessed via the Dictator Game. Approximately 3 days before the onset of the experimental session, participants’ trait empathy was assessed using the Interpersonal Reactivity Index (IRI; Davis, 1983). Consistent with previous findings showing a connection between empathy and prosocial behavior, we hypothesized that participants higher in trait empathy would share more in the Dictator Game than participants lower in empathy. Extending these findings to the domain of stress reactivity, we also hypothesized that stress would increase this gap; stressed individuals high in empathy would share more, and stressed individuals low in empathy would share less, compared to their non-stressed counterparts. Finally, given previous observations that stress effects on social behavior tend to be more pronounced on females than males (Tomova et al., 2014; Duesenberg et al., 2016; Nickels et al., 2017; Youssef et al., 2018), we hypothesized that these effects will be larger in females.

## Materials and Methods

### Participants

Previous reports examining the effects of stress on Dictator Giving to anonymous others reported medium sized effects (von Dawans et al., 2012, 2019). A priori power analysis (ANOVA fixed effects, special main effects and interactions) showed that, with α set to 0.05, a medium effect size f = 0.25, and four groups (male stress, female stress, male control, female control), the required sample size to achieve power (1-β) = 0.80 is 128 participants. We aimed to recruit additional participants to allow for moderating effects of individual differences, however recruitment was halted upon the onset of the Covid-19 pandemic. One hundred and forty six university students (71 male; mean age: 24.5 years, SD = 1.94) participated in the study, which is a part of a broader ongoing study conducted in our lab examining the effects of stress on social behavior. Following the exclusion of 23 participants due to technical problems or prior acquaintance with an experimenter, the final sample size consisted of *n* = 123 (63 male). Exclusion criteria were mental or physical illness, use of medications, and smoking more than five cigarettes per day. Females with irregular menstrual cycles were also excluded from the study. To account for possible effects of hormonal status on stress reactivity, female subjects (*n* = 60) were recruited in three different hormonal statuses. (A) Hormonal Contraceptives: 27 females (stress group *n* = 12) who use hormones-based contraceptives, in days 1–21 of their menstrual cycle, i.e., all days except menstruation. (B) Early-Follicular phase: 17 females (stress group *n* = 7) with a free, regular cycle in days 1–8 of their cycle. (C) Mid-Luteal phase: 17 females (stress group *n* = 9) with a free, regular cycle in days 18–24 of their cycle. Participants were recruited via the university’s online experiment registration system and via social media, and received a fee or credit points in return for their participation. We have reported all measures, conditions, data exclusion, and how we determined our sample sizes.

### Experimental Procedure and Materials

#### Procedure

Following a telephone screen to ensure participants met study criteria, subjects were randomly assigned to either the Trier Social Stress Test (TSST) or the control treatment (described in detail in Section Stress Induction, below). Female subjects were assigned a date for participation in accordance with their self-reported hormonal status. Females with a free cycle were contacted in the days before their participation to ensure their day in the cycle was aligned with the timing of experimental trials. Three days before testing, subjects filled out an online questionnaire battery (see Section Questionnaires Battery). To limit variance in cortisol levels, subjects were instructed to refrain from excessive physical activity and alcohol for 24 h, and from eating and drinking besides water for 120 min, prior to the testing session. In addition, testing commenced on 14:00/16:30 (as circadian cortisol levels are relatively low in the afternoon). Upon arriving to the lab, subjects provided written informed consent, rested for 20 min, and performed two computer-based tasks which will be reported elsewhere (Azulay et al., 2021). Subsequently, the TSST/control session was carried out, immediately followed by the dictator game. Cortisol levels and psychological stress and anxiety were tested throughout the experiment. At the end of the experiment, participants were debriefed and paid.

#### Questionnaires Battery

Participants were assessed via the Interpersonal Reactivity Index (IRI; Davis, 1983), to measure empathy levels. The questionnaire assesses both a total empathy score (computed by summing across all items), and a separate score for each of its four subscale: Empathic Concern—assessing “other-oriented” feelings of sympathy and concern for unfortunate others; Perspective Taking—testing the tendency to spontaneously adopt the psychological point of view of others; Personal Distress—measuring “self-oriented” feelings of personal anxiety and unease in tense interpersonal settings; and Fantasy—evaluating the tendency to transpose oneself imaginatively into the feelings and actions of fictitious characters. Each item in each subscale was rated on a 1–5 scale, asking to what degree the statement describes you, from does not describe me well (1) to describes very well (5).

To ensure that the stress and control group did not differ on other measures which may be associated with stress reactivity, all groups were also assessed for the following: Social Phobia Inventory (SPIN; Connor et al., 2000) to measure social-related anxiety symptoms; Beck’s Depression Inventory (BDI; Beck et al., 1996) to measure depression symptoms; and Demographic details, including socioeconomic status [assessed via standardized self-reported parental economic status (from 1-low to 5-high) and education level] and age.

#### Dictator Game

Participants were given a small box, containing 10 coins of 1 New Israeli Shekel (10 NIS ≈ to 3.11 USD), an envelope, and written instructions. The experimenter left the room after telling them to open the box. The instructions were as follows: “You now receive 10 NIS. You may keep this amount to yourself or divide it however you please between yourself and another student who participated in one of our studies. Put the amount you would like to keep to yourself in your pocket. Put the amount you would like to give to the other in the envelope and seal it”. Participants were then asked to put the envelope back in the box and inside a private tray.

#### Stress Induction

Participants underwent the Trier Social Stress Test (TSST; Kirschbaum et al., 1993), an extensively validated stress procedure shown to elicit a robust cortisol response. The standardized protocol included an anticipatory period (10 min), followed by a public speaking task (5 min) and mental arithmetic task (5 min). The two tasks were performed in front of a panel comprised of one female and one male evaluator who wore lab coats and adopted a non-responsive demeanor. To increase social evaluative threat, participants were informed that their performance would be videotaped and that the evaluators are experts in non-verbal coding. The control treatment was designed to follow as closely as possible the TSST, replicating the instructions and time points, however absent the audience and therefore any social evaluative component in either the speech or mental arithmetic, and employing a simple mental arithmetic task of count up from 1,022 in skips of five (Shalev et al., 2011).

#### Endocrine Stress Response

Salivary cortisol, a reliable measure of HPA axis reactivity, was collected using Salivette swabs (Sarstedt^®^, Nümbrecht-Rommelsdorf, Germany). Measurements occurred at five time points throughout the experiment: −60, −20, +12, +50, +60 min, relative to stress or control treatment onset. Samples were kept on ice during the session, centrifuged at 3,000 rpm at 4°C for 10 min directly following the experimental task, and stored at −70°C. Cortisol concentrations were analyzed via a commercially available ELISA kit (Salimetrics), following the manufacturer’s instructions. Inter-assay coefficient of variation ranged from 6.1 (high control) to 7.5% (low control), and intra-assay coefficient of variation was 5.3%. Changes in cortisol levels were calculated using Area Under the Curve with respect to increase (AUCi), following the procedures described by Pruessner et al. (2003). Note that the Pruessner et al. (2003) paper describes two methods for assessing cortisol reactivity, AUCi and AUCg (Area Under the Curve with respect to ground). We elected to use AUCi rather than AUCg because we were interested in capturing sensitivity to stress, rather than total cortisol output. However, we note that our findings are not dependent on this decision and our robust to either measure.

#### Subjective Stress and Anxiety Measures

Participants rated current subjective stress and anxiety levels on a scale of 1 to 100 via a visual analog scale (VAS; “how much stress/anxiety are you currently experiencing?”). Measurements were performed at three time points (−55, +12, and +65 min, relative to stress onset) reflecting baseline, immediately following stress, and recovery.

### Statistical Analysis

The analysis procedure consists of three parts. The first part validates the effect of the stress manipulation using subjective ratings (self-reported stress and anxiety levels) and salivary cortisol levels. The second part aims to investigate the influence of stress on giving in the dictator game. The last part examines the effect of empathy as well as the empathy x stress interaction on giving levels in the Dictator Game.

In the first part, levels of salivary cortisol and subjective stress and anxiety were used to validate the stress manipulation. Cortisol levels were measured by Area Under the Curve with respect to increase (AUCi). The second cortisol measurement (prior to the stressor) was used as a baseline in the AUCi computation. To evaluate the subjective increase, we computed the average increase in both anxiety and stress ratings during the manipulation (rating post manipulation minus rating pre manipulation) for each participant. A two-way ANOVA model was applied for each stress measure, i.e., cortisol levels and the average increase of subjective ratings. Each model included two factors: treatment (TSST/control) and sex (female/male participants).

In the second part of the analysis, we focused on the Dictator game results. Dictator game results ranged between 0 and 10. Given the censored data, we used Tobit regressions with robust estimators for the analysis (Achtziger et al., 2015). Tobit models assume that there are Dictators who would have given a negative amount, had they not been prevented from doing so due to the experimental design of the game. Previous experiments allowing a take option, show that this is a tenable assumption, and thus Tobit models are often preferred over OLS models when analyzing Dictator Game allocations (Engel, 2011). We first examined the effects of stress and empathy on giving separately. To address the stress effect, we examined a model including treatment and sex, and their interaction. To further evaluate the role of cortisol reactivity in contributing to differences in Dictator Game giving, we reanalyzed the model while replacing the binary treatment factor with scaled AUCi values (centered and standardized to mean = 0 and SD = 1), a continuous variable representing cortisol reactivity from baseline. Then, to test the effect of empathy on giving we examined a model which includes the effects of sex, empathy, the interaction between them, and with treatment as a covariate.

Finally, we examined whether differences in Dictator giving could be explained by the combined stress x empathy interaction. Therefore, we added an empathy factor to the ANOVA model containing treatment and sex. The model contained all the interactions between the three factors. To provide greater resolution as to the source of the empathy effect, we next performed the same analysis for each subscale of the IRI empathy questionnaire separately. All IRI values (total and subscales) were centered and standardized (mean = 0 and SD = 1). To further explore models with significant effects among female participants, we also applied models which include hormonal status as covariate in the female only sub-sample. Tobit models were analyzed using Stata (StataCorp, 2021, Release 17), all other tests were computed using R version (4.0.2).

## Results

### Descriptive Statistics of Subjects’ Characteristics

The TSST and control groups did not differ on their scores for empathy (IRI), social anxiety (SPIN) depression (BDI) or socioeconomic status (see Supplementary Table 1). The internal consistencies of the three questionnaires, measured by Cronbach’s alpha, were acceptable (IRI: α = 0.74, SPIN: α = 0.8, BDI: α = 0.91).

### Validation of Stress Induction Procedure

We used levels of salivary cortisol, as well as subjective stress and anxiety measures, to validate that the stress manipulation caused an increase in the physiological and psychological stress response.

#### Salivary Cortisol Levels

As in previous studies, the TSST procedure successfully increased participants’ salivary cortisol levels. A two-way ANOVA was conducted on cortisol levels. The model included treatment (TSST/control) and sex (female/male) as factors. We found the expected treatment effect [*F*_(1,119)_ = 42.04., *p* < 0.001, $\eta_{p}^{2}$ = 0.26], whereby cortisol levels increased following manipulation onset in the TSST treatment but not in the control treatment. The model also revealed a significant interaction between treatment and sex [*F*_(1,119)_ = 7.49, *p* = 0.007, $\eta_{p}^{2}$ = 0.06]. Males showed a significantly higher cortisol increase compared to that of females, in line with previous studies (Liu et al., 2017).

#### Subjective Stress and Anxiety Levels

Similar to salivary cortisol levels, subjective stress and anxiety levels were increased by the TSST treatment, but not by the control treatment. We found a significant treatment effect for the average increase of subjective stress and anxiety [*F*_(1,119)_ = 17.37, *p* < 0.001, $\eta_{p}^{2}$ = 0.13]. Main effect of and interaction with sex were not significant (*p* > 0.13), reflecting a similar subjective stress and anxiety response in females and males.

### Effects of Stress and Empathy on Dictator Game Giving

#### No Main Effect of Stress on Dictator Game Giving

**FIGURE 1:**
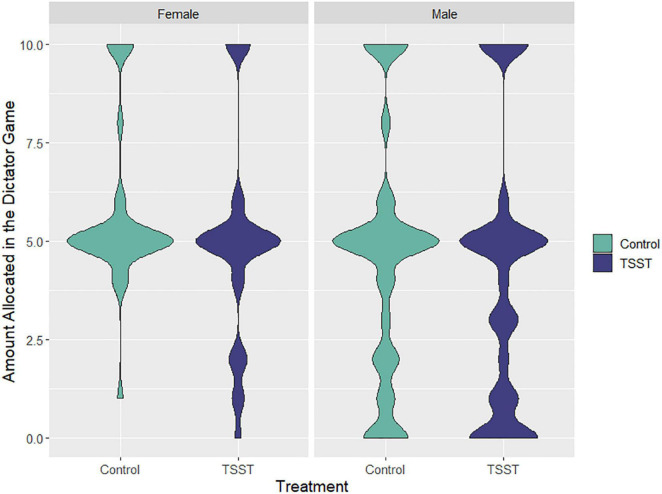
Violin Plots of the amount of money allocated to others by female and male participants in the dictator game. Amount of money allocated was smoothed using a gaussian kernel with bandwidth of 0.3 SD. The graphs on the left depict female participants and the graphs on the right depict results for male participants. Participants in the control group are shown in cyan, while stressed participants are shown in purple.

As a first step, we examined whether males’ and females’ pro-social behavior, as measured by the level of giving in the Dictator Game, was influenced by stress exposure. We applied a Tobit model for both female and male participants, including a treatment factor (TSST/control) and a sex factor (male/female). While we observed a marginal main effect of sex (*t* = −1.72, *p* = 0.088) on Dictator giving, indicating that females allocated more money to others compared to males, the stress treatment (*t* = −1.12, *p* = 0.264) and the interaction between sex and treatment (*t* = 0.13, *p* = 0.897) were not significant (see Figure 1).

Next, to assess if rather than stress exposure, measured levels of cortisol reactivity could account for Dictator giving levels, we replaced the binary treatment variable with a continuous measure of cortisol reactivity (AUCi). This model revealed a significant effect of sex (*t* = −2.1, *p* = 0.038), however, again there was no effect of treatment (*t* = 0.64, *p* = 0.522) or interaction between sex and treatment (*t* = −0.99, *p* = 0.324).

#### Main Effects of Empathy on Dictator Game Giving

To examine the association between empathy and the amount of money allocated to others in the Dictator Game, we used a Tobit model with two factors: Sex (male/female) and empathy score (measured by the IRI questionnaire). Because females showed higher levels of empathy compared to males [*t*_(119.8)_ = 3.47, *p* = 0.0007], in line with previous findings (Eckel and Grossman, 1998), all analyses examining empathy effects on giving levels controlled for sex (the models include the factors of sex, empathy and interaction between sex and empathy). Additionally, to account for possible stress related effects, we included the stress continuous variable (AUCi) as a covariate. We find a marginal significant effect of empathy [b = 0.75, CI (−0.14–1.63), *p* = 0.098], suggesting that participants scoring higher in empathy levels allocated more money to others than participants with relatively lower empathy levels.

#### Interaction Effect of Stress and Empathy on Dictator Game Giving

We next add the stress X empathy term to the model to test the interactive effect of empathy and stress on Dictator giving. Here too, all analyses controlled for sex (including the interactions of sex with all others factor as well). The first Tobit model included the binary treatment factor. The model did not reveal any significant results [treatment: *b* = −0.98 CI (−2.55–0.60), *p* = 0.223; sex: *b* = −1.30 CI (−3.03–0.42), *p* = 0.138; empathy: *b* = 0.12 CI (−0.62–0.86), *p* = 0.751; treatment X sex: *b* = 0.48 CI (−2.18–3.13), *p* = 0.724; treatment X empathy: *b* = 1.22 CI (−0.51–2.94), *p* = 0.166, sex X empathy: *b* = 0.38 CI (−1.07–1.84), *p* = 0.602, treatment X sex X empathy: *b* = −0.27 CI (−2.82–2.29), *p* = 0.836]. Next, we replaced the binary treatment factor with cortisol AUCi levels (see full statistics in Table 1). This Tobit model revealed three significant effects: A three-way interaction between empathy, AUCi and sex [b = −1.71 CI (−3.05—0.37), *p* = 0.013], an interaction between empathy and AUCi [*b* = 1.99 CI (0.88–3.09), *p* = 0.001] and a main effect of empathy [*b* = 1.00 (0.20–1.80), *p* = 0.014]. The significant interaction between empathy and AUCi shows that participants with relatively higher empathy levels have a positive correlation between AUCi and Dictator giving, while the pattern within participants with lower empathy levels is in the opposite direction, namely less empathetic individuals gave less money in the Dictator Game under stress. The main effect of empathy shows that participants with relatively higher levels of empathy allocated more money to others in general.

**TABLE 1:** Cortisol area under the curve with respect to increase (AUCi), sex, and empathy measure effects on giving in the dictator game.

	(A) IRI empathy questionnaire total score			(B) Empathic concern subscale			(C) Perspective taking subscale			(D) Personal distress subscale			(E) Fantasy subscale		
	B	SE	*p*-value	B	SE	*p*-value	B	SE	*p*-value	B	SE	*p*-value	B	SE	*p*-value
Cortisol AUCi	−0.25	0.28	0.38	−0.72	0.59	0.22	−0.23	0.88	0.79	0.31	0.61	0.61	0.51	0.46	0.27
Sex (male)	−0.85	0.68	0.22	−0.82	0.68	0.23	**−1.31**	**0.66**	**0.05**	**−2.01**	**0.73**	**0.007**	**−**1.29	0.68	0.06
Empathy Measure	**1.00**	**0.40**	**0.01**	**1.03**	**0.34**	**0.003**	0.63	0.38	0.10	**−**0.36	0.47	0.45	0.71	0.44	0.11
Cortisol AUCi * sex	0.08	0.54	0.89	0.47	0.76	0.54	**−**0.55	1.00	0.59	**−**0.88	0.75	0.24	**−**0.70	0.70	0.32
Cortisol AUCi * empathy measure	**1.99**	**0.56**	**0.001**	**1.47**	**0.60**	**0.02**	1.00	0.84	0.24	**−**0.13	0.93	0.89	1.11	0.61	0.07
Sex * empathy measure	−0.10	0.64	0.87	−0.24	0.63	0.70	1.07	0.65	0.10	**−**0.73	0.77	0.34	**−**0.10	0.74	0.89
Cortisol AUCi * sex * empathy measure	**−1.71**	**0.68**	**0.01**	**−1.53**	**0.69**	**0.03**	0.56	1.10	0.61	**−**0.79	1.02	0.45	**−**0.62	0.85	0.46

*Every three columns represent a different scale for IRI empathy questionnaire—(A) Total score, (B) Empathic concern, (C) Perspective taking, (D) Personal distress, and (E) Fantasy. Presented are coefficient estimates (betas), standard errors, and p-values of each factor. Sex is a dummy variable with female as baseline. Significant results are presented in bold.*

**FIGURE 2:**
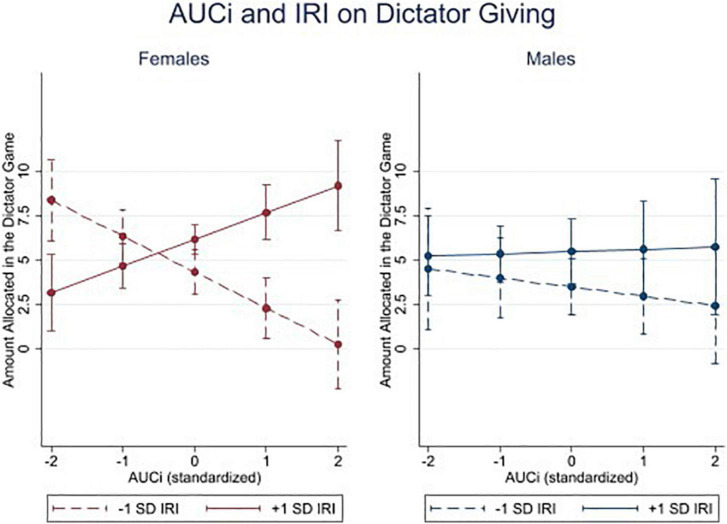
Area under the curve with respect to increase (AUCi) and interpersonal reactivity index (IRI) on dictator giving. Amount transferred in the dictator game as a function of cortisol reactivity (Area under the curve with respect to increase, standardized to mean = 0 and SD = 1). The figure shows the predicted amount transferred in the Dictator Game for females (left, *N* = 60) and males (right, *N* = 63). Lines represent marginal means for individuals 1 SD above (solid) or below the mean (dashed) in empathy. Bars represent 95% confidence intervals.

To further examine the three-way interaction, and specifically to test whether the interaction between AUCi and empathy on Dictator giving differed between males and females, we performed the same analysis for females and males separately. We revealed that although both models show a similar pattern of a main effect of empathy and an interaction between AUCi and empathy on Dictator giving, these effects are significant in the females’ model [empathy: *b* = 0.93 CI (0.20–1.65), *p* = 0.014; interaction: *b* = 1.77 CI (0.84–2.70), *p* < 0.001], but not in the males’ model [Empathy: b = 1.00 CI (−0.15–2.15), *p* = 0.086; interaction: *b* = 0.32 CI (−0.56–1.21), *p* = 0.467, see Figure 2]. The main effect of AUCi was not significant in either model [Female: *b* = −0.26 CI (−0.78–0.26), *p* = 0.323; Males: *b* = −0.20 CI (−1.25–0.86), *p* = 0.712], as in the model which include both sexes.

Females with low cortisol responses generally fall into two groups. They either were in the control condition, in which case they were not stressed. Or they were in the TSST condition, but did not show a pronounced cortisol response to the stressor. When examining the two subgroups, we find that in the control condition, the IRI score is not predictive of dictator giving. Tobit regressions of standardized IRI scores predicting Dictator Giving for females in the control condition was positive, but not statistically significant [b(zIRI) = 0.11 CI (−0.61–0.84), *p* = 0.76], suggesting that in our data, under non-stressful conditions, IRI empathy scores are not a robust indicator of dictator giving for females. In contrast, under the TSST condition, IRI scores for females are positively associated with Dictator Giving, however are only marginally significant [b(zIRI) = 1.3 CI (−0.23–2.78), *p* = 0.09]. Moreover, examining cortisol reactivity (AUCi) within the female TSST group shows a significant interaction between IRI and AUCi on Dictator Giving [B(interaction) = 2.24 CI (0.58–3.89), *p* = 0.01]. This suggests that the more selfish behavior by individuals scoring higher in the IRI is driven primarily by females who show little to no cortisol reactivity when exposed to psychosocial stress, rather than by empathetic females showing being selfish under non-stressful conditions.

Finally, we tested whether hormonal status influenced the females’ model by adding hormonal status as a covariate. We found that hormonal status did not change the significance of empathy and the interaction between AUCi and empathy (For full statistics see Supplementary Table 2).

To further investigate the interaction between stress and empathy on Dictator Game giving, we performed the same analysis with each of the IRI empathy questionnaire subscales–empathic concern, perspective tasking, personal distress and fantasy. The model including the empathic concern subscale showed a similar pattern effect as in the total IRI score model (main effect of empathy, interaction between empathy and AUCi and a 3-way interaction of empathy, AUCi and sex). The models including perspective taking and personal distress revealed only a significant effect of sex, and the Fantasy did not reach significance for any factor. For full statistics see Table 1.

## Discussion

Our results show that effects of acute stress on social behavior are moderated by sex and trait empathy. Consistent with previous findings (Berger et al., 2016), these effects are most prominent when examining change in cortisol, rather than the binary stress vs. non-stress (control) exposure measure, suggesting that the magnitude of the cortisol response may be a key factor. While the stress task is designed to elicit a stress response evoked by the social evaluative, uncontrollable nature of the task, and the control task does not, there are nevertheless individual differences in cortisol reactivity, with some non-responders in the stress task, and some responders in the control task see Miller et al. (2013) for classification criteria]. Consequently, it is common practice to use the AUCi measure, which captures this variation in stress reactivity. For females high in empathy, greater cortisol reactivity resulted in more generous behavior toward an unfamiliar other. In contrast, for females low in empathy, greater cortisol reactivity resulted in less generous behavior. A similar trend, despite being non-significant, was shown in males.

Interestingly, a recent study (Zhang et al., 2019) also found that empathy levels (i.e., Empathic Concern subscale) moderated the link between cortisol reactivity and generosity. However, they reported a different directionality, whereby higher cortisol reactivity was associated with less generosity. This may be due to the different stress procedures employed—with Zhang et al. using the group version of the TSST (von Dawans et al., 2011), while we employed the one-person TSST version. These disparate paradigms have been shown to differentially affect participants’ subjective experience (Steinbeis et al., 2015); for example, the group setting instills greater social comparisons, while the individual setting is typically thought to lead to greater feelings of isolation as well as greater cortisol reactivity, which may differentially affect both experienced empathy and social preferences across the two studies (Vors et al., 2018).

Our findings bear consequences to ongoing discussions characterizing social responses to stress as befitting either the Fight, Flight or Freeze vs. Tend and Befriend theories. Individual differences in empathy may serve as a useful proxy for discerning which of the models is more appropriate, particularly for females. Adding information regarding trait empathy accounts for previously unexplained heterogeneity in Dictator giving in response to stress. While the specific biological mechanisms underpinning these effects are not currently well-understood, exposure to stress leads to secretionl of the hormone oxytocin (Nishioka et al., 1998; de Jong et al., 2015), which may faciliate more prosocial responses in individuals high in empathy. Future research will need to determine if empathy also plays an important role in modulating the effects of stress on social behavior in other decision-making paradigms (e.g., trust game, ultimatum game, etc.).

Another aspect for future research of stress effects on sociality is the target’s identity. The Tend and Befriend model, for example, relates to prosocial, affiliative behaviors toward “others” under stressful circumstances, but are all others treated the same? One possible clue to this question can be found in Margittai et al. (2015) findings, showing that generosity seems to be particularly pronounced toward socially close (as opposed to distant) individuals. While the current study showed that in the one-shot Dictator game, when the identity of the recipient is anonymous, stress does not directly affect giving rates, other studies may benefit from exploring variations to the protocol. For example, if and how different degrees of social distance—the degree to which a person feels close to or far from another person—affects generosity levels (Jones and Rachlin, 2006).

More generally, our findings are consistent with the deliberation-to-intuition (SIDI) model proposed by Yu (2016)–see introduction, which posits that under stressful circumstances, deliberative decision-making processes are less available. Rather, individuals react in accordance to their basic intuition, or in this case in a manner more consistent with their personality. If they are dispositionally more empathetic, their stress-induced cortisol reactivity will direct them toward greater prosocial behavior, and if they have lower levels of trait empathy, stress will decrease prosocial behavior.

Our study presents some limitations. We used the Dictator Game paradigm to assess levels of generosity. While this procedure is extensively researched and used, employing other decision-making paradigms may have allowed a broader understanding of the relations between acute stress, empathy and social decision making. For example, future studies may benefit from assessing real-life generosity and sharing behaviors (e.g., donation behavior), or by including additional games which assess related phenotypes (e.g., altruistic punishment, trust, etc.). As both lab-based and more ecological tasks have their advantages and disadvantages, employing several tasks from different domains may provide a more nuanced understanding of the effects of stress on different types of social behavior. Additionally, the Dictator Game stake in the current study was 10 New Israeli Shekel, and it was recently shown that the higher the stakes, the lower the generosity (Larney et al., 2019). To further generalize the current findings, future studies may set different stakes, which will assist in determining the link between stress, empathy, and perhaps, stake-dependent generosity. In addition, the current study did not measure empathy levels throughout the procedure, and therefore cannot tell if current empathy levels (as opposed to trait empathy) were impacted by our design or affected the results. As mentioned above, former evidence is conflicted on the subject, and while there is some evidence that empathy levels might change as a function of stress (Zilioli et al., 2015), other studies (Duesenberg et al., 2016) suggest that it does not.

The current study contributes to our understanding of stress and social behavior and provides evidence that sex and individual levels of trait empathy play a key role in determining levels of generosity under stressful, cortisol-eliciting circumstances. In the context of the ongoing debate comparing the Freeze, Fight or Flight, and Tend and Befriend models, our findings suggest that there is room to accommodate both models. The social response to stress is not monotonic, but rather depends on specific contexts. As previously shown, stressor nature, its duration, and the type of social task directs social preferences in response to stress [see a review in von Dawans et al. (2021)]; And here, we suggest that in addition to sex, levels of cortisol reactivity interact with individual differences in empathy to also play a key role in determining social behavior.

The characterization of trait empathy as reflecting a more broad-based biobehavioral strategy for coping with stress has important implications both for research on empathy, and for research on coping with stress. If people high in empathy become more prosocial under stress, and (speculatively) show greater social motivation (to request and receive social support), while people low in empathy become less prosocial and shy away from others, this places trait empathy as a key feature in directing availability and receipt of social support under times of stress. Importantly, social support during stress has consistently been found to have profound effects on health and well-being (Ozbay et al., 2007), and this research begins to tease apart the mechanisms which may facilitate such behaviors.

To conclude, the current study suggests that social preferences, such as the amount people choose to give in a one-shot Dictator Game, is moderated by both acute stress exposure and trait empathy. We show that highly empathic females in particular are prone to be more altruistic in cortisol-eliciting stressful circumstances. In the context of research on evolutionary perspectives of stress and sociality (i.e., the Fight or Flight and Tend and Befriend models), we therefore suggest that turning a spotlight toward the effects of individual differences in traits and personal characteristics will allow a more nuanced and precise description of the effect of acute stress on how people interact with others.

## Data Availability Statement

The datasets presented in this study can be found in online repositories. The names of the repository/repositories and accession number(s) can be found below: Data and code for the studies are available on OSF (https://osf.io/j5d3e/?view_only=4da950ee918d41129a152180285d538f).

## Ethics Statement

The studies involving human participants were reviewed and approved by Ethics Committee of the Faculty of Social Sciences at The Hebrew University of Jerusalem. The patients/participants provided their written informed consent to participate in this study.

## Author Contributions

HA ran the experimental sessions. HA, NG, and SI analyzed the data. YP and SI provided funding. All authors designed the experiment, wrote, reviewed, and edited the manuscript, and approved the final version of the manuscript for submission.

## Conflict of Interest

The authors declare that the research was conducted in the absence of any commercial or financial relationships that could be construed as a potential conflict of interest.

## Publisher’s Note

All claims expressed in this article are solely those of the authors and do not necessarily represent those of their affiliated organizations, or those of the publisher, the editors and the reviewers. Any product that may be evaluated in this article, or claim that may be made by its manufacturer, is not guaranteed or endorsed by the publisher.
