# Supplementary material for: Empathy Modulates the Effect of Stress Reactivity on Generous Giving
Source: Front Neurosci. 2022 Apr 25;16:814789. doi: 10.3389/fnins.2022.814789 (PMC9081844; doi:10.3389/fnins.2022.814789)
Supplement: Supplementary file 1 [file Table_1.DOCX]

Supplementary material

1. **Descriptive statistics of subjects’ characteristics**

To test whether the TSST and control group differed in each of the measure in the questionnaire battery, we used t-test to compare between the groups on each trait. We did not assume equal variance and the degree of freedom was adjusted with a Welch correction. We did not find any significant differences between treatment and control groups on the any of the questionnaires assessed in the study.

|  | Mean Control | Mean TSST | DF after Welch correction | t-value | p-value |
| --- | --- | --- | --- | --- | --- |
| IRI | 96.95 | 95.14 | 119.8 | 0.92 | 0.36 |
| BDI | 6.42 | 5.33 | 113.41 | 1.17 | 0.25 |
| SPIN | 16.48 | 15.6 | 120.65 | 0.39 | 0.69 |
| SES | - 0.07 | 0.074 | 112.95 | - 0.78 | 0.44 |

**Supplementary Table 1: Descriptive Statistics and t-tests comparing control and TSST groups for each questionnaire.**

1. **Females’ model with hormonal status as a covariate**

To further examine the interaction between AUCi and empathy within females, we added the hormonal status (contraceptives, early-follicular and mid-luteal) as a covariate. Including the hormonal status in our model did not change statistical inferences for the main effect of empathy on Dictator giving or for the interaction between AUCi and empathy (either for the total IRI score or for the empathic concern subscale).

|  | (A) IRI Empathy Questionnaire Total Score | | | (B) Empathic Concern  Subscale | | |
| --- | --- | --- | --- | --- | --- | --- |
|  | B | SE | p-value | B | SE | p-value |
| Cortisol AUCi | -.14 | 0.23 | 0.53 | -0.478 | 0.52 | 0.37 |
| Empathy Measure | **0.91** | **0.36** | **0.01** | **0.91** | **0.30** | **0.004** |
| Cortisol AUCi * empathy measure | **1.59** | **0.45** | **0.001** | **1.11** | **0.53** | **0.04** |
| Hormonal status (1) | -0.18 | 0.78 | 0.82 | -0.36 | 0.84 | 0.67 |
| Hormonal status (2) | 0.90 | 0.83 | 0.28 | 0.90 | 0.88 | 0.31 |

**Supplementary Table 2: Cortisol AUCi and IRI empathy questionnaire effects on generosity in the Dictator Game while taking into consideration the hormonal status**. Every three columns represents a different model for IRI empathy questionnaire – total score and Empathic Concern. Presented are coefficient estimates (betas), standard errors, and p-values of each factor. Hormonal status is a dummy variable with the contraceptives group as a baseline. Hormonal status (1) represents the addition of the females in the early-follicular phase and hormonal status (2) represents females in the mid-luteal phase of the menstrual cycle.
